# CCL2 Expression in Tumor Cells and Tumor-Infiltrating Immune Cells Shows Divergent Prognostic Potential for Bladder Cancer Patients Depending on Lymph Node Stage

**DOI:** 10.3390/cancers12051253

**Published:** 2020-05-15

**Authors:** Markus Eckstein, Elena Epple, Rudolf Jung, Katrin Weigelt, Verena Lieb, Danijel Sikic, Robert Stöhr, Carol Geppert, Veronika Weyerer, Simone Bertz, Astrid Kehlen, Arndt Hartmann, Bernd Wullich, Helge Taubert, Sven Wach

**Affiliations:** 1 Institute of Pathology, University Hospital Erlangen, FAU Erlangen-Nürnberg, 91054 Erlangen, Germany; markus.eckstein@uk-erlangen.de (M.E.); elena.epple@fau.de (E.E.); Rudolf.Jung@uk-erlangen.de (R.J.); robert.stoehr@uk-erlangen.de (R.S.); carol.geppert@uk-erlangen.de (C.G.); veronika.weyerer@uk-erlangen.de (V.W.); simone.bertz@uk-erlangen.de (S.B.); Arndt.Hartmann@uk-erlangen.de (A.H.); 2 Department of Urology and Pediatric Urology, University Hospital Erlangen, FAU Erlangen-Nürnberg, 91054 Erlangen, Germany; Katrin.Weigelt@uk-erlangen.de (K.W.); Verena.Lieb@uk-erlangen.de (V.L.); danijel.sikic@uk-erlangen.de (D.S.); Bernd.Wullich@uk-erlangen.de (B.W.); sven.wach@uk-erlangen.de (S.W.); 3 Institute of Medical Microbiology, Medical School, Martin-Luther-University Halle-Wittenberg, 06112 Halle (Saale), Germany; astrid.kehlen@uk-halle.de

**Keywords:** CCL2, bladder cancer, prognosis, tumor cells, immune cells, lymph node stage

## Abstract

Bladder cancer (BCa) is the ninth most commonly diagnosed cancer worldwide. Although there are several well-established molecular and immunological classifications, markers for tumor cells and immune cells that are associated with prognosis are still needed. The chemokine CC motif ligand 2 (CCL2) could be such a marker. We analyzed the expression of CCL2 by immunohistochemistry (IHC) in 168 muscle invasive BCa samples using a tissue microarray. Application of a single cut-off for the staining status of tumor cells (TCs; positive vs. negative) and immune cells (ICs; ≤6% of ICs vs. >6% of ICs) revealed 57 cases (33.9%) and 70 cases (41.7%) with CCL2-positive TCs or ICs, respectively. IHC results were correlated with clinicopathological and survival data. Positive CCL2 staining in TCs was associated with shorter overall survival (OS), disease-specific survival (DSS), and relapse-free survival (RFS) (*p* = 0.004, *p* = 0.036, and *p* = 0.047; log rank test) and appeared to be an independent prognostic factor for OS (RR = 1.70; *p* = 0.007; multivariate Cox’s regression analysis). In contrast, positive CCL2 staining in the ICs was associated with longer OS, DSS, and RFS (*p* = 0.032, *p* = 0.001, and *p* = 0.001; log rank test) and appeared to be an independent prognostic factor for DSS (RR = 1.77; *p* = 0.031; multivariate Cox’s regression analysis). Most interestingly, after separating the patients according to their lymph node status (N0 vs. N1+2), CCL2 staining in the ICs was differentially associated with prognosis. In the N0 group, CCL2 positivity in the ICs was a positive independent prognostic factor for OS (RR = 1.99; *p* = 0.014), DSS (RR = 3.17; *p* = 0.002), and RFS (RR = 3.10; *p* = 0.002), whereas in the N1+2 group, CCL2 positivity was a negative independent factor for OS (RR = 3.44; *p* = 0.019)) and RFS (RR = 4.47; *p* = 0.010; all multivariate Cox’s regression analyses). In summary, CCL2 positivity in TCs is a negative prognostic factor for OS, and CCL2 can mark ICs that are differentially associated with prognosis depending on the nodal stage of BCa patients. Therefore, CCL2 staining of TCs and ICs is suggested as a prognostic biomarker for BCa patients.

## 1. Introduction

Bladder cancer (BCa) is the 9th most commonly diagnosed cancer and the 13th leading cause of cancer-related death worldwide [1]. It originates from the urothelium of the ureter, urethra (upper urinary tract), and bladder [1,2]. Clinical management of BCa [2,3], etiology, and diagnostic, prognostic, or predictive biomarkers for BCa have been described extensively [4,5]. Treatment options are available for both superficial and invasive BCa; however, metastatic disease still presents a serious clinical problem with limited therapeutic options. Interestingly, BCa and breast cancer can be subdivided into basal and luminal subtypes that harbor prognostic and predictive relevance [6,7,8,9,10,11,12,13]. However, this classification is mostly based on RNA expression levels. Recently, Sjödahl et al. proposed an algorithm for immunohistochemical (IHC) subtyping of muscle-invasive bladder cancers [14]. In addition, there are several classifications by the presence of immune cells. These classifications are the NLR (neutrophil-lymphocyte ratio), PLR (platelet-lymphocyte ratio), CAR (C-reactive protein/albumin ratio), and SII (systemic immune-inflammation index: platelet count × neutrophil count/lymphocyte count) [15,16,17,18]. Several inflammatory biomarkers and their association with BCa prognosis have been described (reviewed in [19]). For example, the abundance of cytotoxic T lymphocyte markers, such as CD3 and CD8, was associated with improved survival, while the macrophage marker CD68 was associated with worse survival, recurrence, and progression in bladder cancer [19,20]. Recently, an association between immune cells, such as CD8 tumor-infiltrating lymphocytes, in the tumor microenvironment and the survival of BCa patients was further confirmed [21,22]. In addition, the molecular characteristics of immune cells were considered by an immune classification based on T cell infiltration, as in the TMIT classification (tumor microenvironment immune type: PD-L1 and CD8) or a classification published by our group with a unique immune evasion phenotype with constitutive overexpression of PD-L1 on tumor cells [23,24]. However, it would be helpful to identify a few or a single protein marker expressed in tumor cells and immune cells that is associated with prognosis in BCa.

An interesting candidate for marking both tumor cells and immune cells at the protein level is the chemokine CCL2 (chemokine, CC motif, ligand 2; synonym monocyte chemotactic protein 1 (MCP-1)). CCL2 production in tumors facilitates the accumulation of immune-suppressive tumor-promoting/tumor-associated macrophages, but CCL2 can also activate monocytes for tumor cell killing [25]. The role of CCL2 in different tumors is controversial [25,26]. High levels of CCL2 have been associated with cancer progression in several types of cancer, including breast, prostate, colorectal, kidney, and thyroid cancers [27,28,29,30,31,32]. Recently, Chen et al. showed that high CCL2 mRNA expression in a TCGA cohort of bladder cancer patients was associated with poor OS and DFS [33]. However, according to human protein atlas, CCL2 protein is not detected in nonmalignant urothelium. However, BCa patients with a higher tumor stage, higher tumor grade, or metastasis have a significantly higher CCL2 concentration in their urine than patients with a lower tumor stage, with a lower tumor grade, or without metastasis [34]. On the other hand, in resected pancreatic cancer patients, CCL2 is an independent favorable prognostic factor with indirect effects on pancreatic cancer cell growth via cytokines, such as IL1B [35]. Furthermore, in soft tissue sarcoma patients, high CCL2 mRNA expression is associated with a good prognosis [36], and higher CCL2 expression was significantly associated with better OS in non-small-cell lung cancer (NSCLC) patients [37]. In addition, a protective role of the CCR2/CCL2 chemokine pathway has been demonstrated by cytotoxic T lymphocytes’ migration toward melanoma cells that secrete CCL2, which results in tumor cell apoptosis [38], and further by the recruitment of subsets of tumor-infiltrating lymphocytes (type 1 cytotoxic gamma-delta T lymphocytes) to tumor beds in a melanoma model [39]. The aim of our study was to analyze for the first time the expression of CCL2 in BCa tumor cells and immune cells by immunohistochemistry and to correlate the results with clinicopathological and survival data to assess the impact of CCL2 as a prognostic factor in BCa.

## 2. Results

### 2.1. CCL2 Expression and Correlation with Clinicopathological Parameters and Expression of Selected Proteins

**Figure 1:**
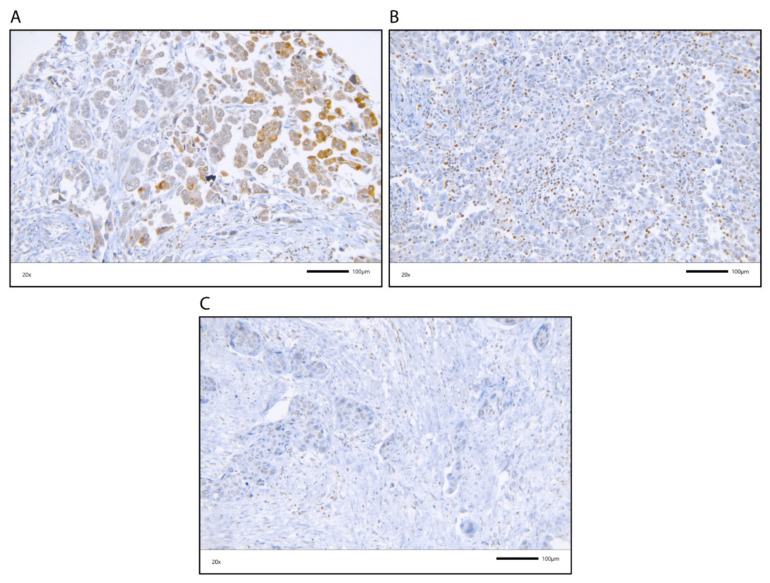
CCL2 immunohistochemical staining in TCs and ICs. Upper row, (**A**) TCs, positive (ICs, negative); (**B**) Positive ICs with >6% CCL2 positivity (TCs, negative); Lower row, (**C**) TCs and ICs, negative; All photos are at 20× magnification.

A cohort of 168 muscle invasive BCa patients was studied for their CCL2 protein expression by immunohistochemistry (IHC) (Figure 1 and Table 1). The clinicopathological data of the muscle invasive BCa patients are summarized in Table 1. CCL2 protein expression was analyzed in tumor cells (TCs; N = 168) and in immune cells (ICs; N = 168). CCL2 expression was scored as positive vs. negative for TCs and as a percentage of CCL2-positive cells for ICs in the tumor cell area. We detected 111 cases (66.1%) and 98 cases (58.3%) with no CCL2-stained TCs or ICs and 57 cases (33.9%) and 70 cases (41.7%) with CCL2-positive TCs or ICs, respectively (Supplementary Table S1). CCL2 protein expression detected by IHC is shown in Figure 1.

Next, we analyzed whether CCL2 staining was associated with clinicopathological and molecular parameters by correlation tests (Spearman’s bivariate correlation test).

In TCs, there was no association of CCL2 staining with gender, tumor stage, PD-L1 expression in ICs or TCs, or with CCL2 staining in ICs. A significant positive association was detected with age (r_s_ = 0.240; *p* = 0.002). A significant negative correlation was observed for CCL2 staining and overall survival (OS) (r_s_ = −0.242; *p* = 0.002) and recurrence-free survival time (r_s_ = −0.199; *p* = 0.010; Supplementary Table S2).

There was no association of the CCL2-positive IC percentage with age, gender, or CCL2 staining in TCs. A significant positive association was found for the CCL2-positive IC percentage and OS (r_s_ = 0.177; *p* = 0.022), disease-specific survival (DSS) (r_s_ = 0.344; *p* < 0.001), recurrence-free survival (RFS) (r_s_ = 0.353; *p* < 0.001), CK5 (r_s_ = 0.201; *p* = 0.009), percentage of stromal TILs (r_s_ = 0.559; *p* < 0.001), CD3 (r_s_ = 0.604; *p* < 0.001), CD8 (r_s_ = 0.591; *p* < 0.001), CD68 (r_s_ = 0.500; *p* < 0.001), PD-L1 expression on ICs (r_s_ = 0.541; *p* < 0.001), and PD-L1 expression on TCs (r_s_ = 0.351; *p* < 0.001). A negative correlation for the CCL2-positive IC percentage was detected with tumor stage (r_s_ = −0.155; *p* = 0.045), lymph node stage (r_s_ = −0.210; *p* = 0.006), and molecular subtype (r_s_ = −0.336; *p* < 0.001; Supplementary Table S2).

### 2.2. Association of CCL2 Protein Expression in TCs and Survival

**Figure 2:**
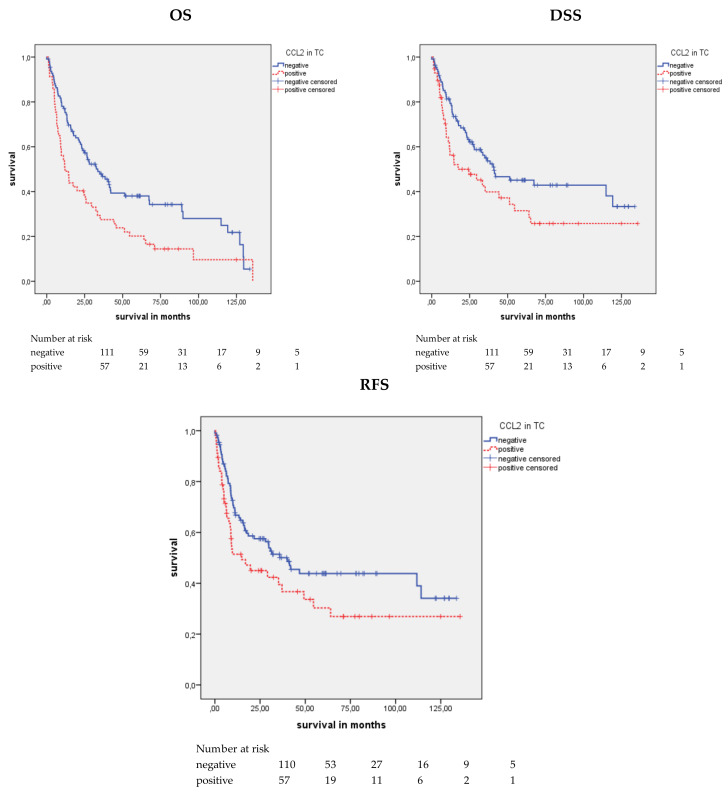
Kaplan–Meier analysis: Association between CCL2 expression in TCs and prognosis. Positive CCL2 expression in TCs was associated with a shorter mean OS (*p* = 0.004), mean DSS (*p* = 0.036), and mean RFS (*p* = 0.047) than negative CCL2 expression.

CCL2 staining in the TCs was considered positive or negative. A significant association between positive CCL2 staining and a shorter mean OS (*p* = 0.004), mean DSS (*p* = 0.036), and mean RFS (*p* = 0.047) was detected in the Kaplan–Meier analysis (log rank test) (Table 2 and Figure 2). When comparing the patients with CCL2-positive TCs with those with CCL2-negative TCs, the mean OS was 33.6 months vs. 55.4 months, the mean DSS was 49.9 vs. 67.9 months, and the mean RFS was 48.3 vs. 65.2 months. In the univariate Cox’s regression analysis, CCL2 positivity was associated with a 1.69-fold increased risk of death (*p* = 0.005), a 1.57-fold increased risk of disease-specific death (*p* = 0.037), and a 1.53-fold increased risk of recurrence (*p* = 0.047; Table 3). In multivariate Cox’s regression analysis (adjusted for tumor stage, lymph node stage, molecular subtype), CCL2 positivity appeared to be an independent poor prognostic factor only for OS (RR (relative risk) = 1.70; *p* = 0.007; Table 3).

Next, we analyzed the association of CCL2 expression in TC with prognosis (OS, DSS, RFS) in different patient subgroups (Table 2 and Table 3). In the Kaplan–Meier analysis, positive CCL2 expression in TC was associated with shorter OS in the subgroups: pT2 (*p* = 0.003), the N0 (*p* = 0.016), not with chemotherapy-treated (*p* = 0.025), with chemotherapy-treated patients (*p* = 0.043), and the molecular subtype luminal (*p* = 0.010). In addition, in the Kaplan–Meier analysis, positive CCL2 expression in TC was associated with a shorter DSS in the subgroup with chemotherapy-treated patients (*p* = 0.045). Accordingly, we found in a univariate Cox’s regression analysis an increased risk of death in the subgroups: pT2 (RR = 3.28; *p* = 0.003), the N0 (RR = 1.83; *p* = 0.018), patients without adjuvant chemotherapy (RR = 1.62; *p* = 0.026), patients with adjuvant chemotherapy (RR = 2.05; *p* = 0.048), and the molecular subtype luminal (RR = 2.05; *p* = 0.012). Furthermore, CCL2 positivity in TC was associated with a 2.10-fold increased risk of tumor-associated death (*p* = 0.050) for patients with adjuvant chemotherapy. In multivariate Cox’s regression analysis (adjusted for tumor stage, lymph node stage, molecular subtype), CCL2 positivity remained an independent prognostic marker for OS in the subgroups: pT2 (RR = 4.02; *p* = 0.002), patients with adjuvant chemotherapy (RR = 2.56; *p* = 0.018), and in the molecular subtype luminal (RR = 1.97; *p* = 0.021), and an independent prognostic marker for DSS in the subgroup of patients with adjuvant chemotherapy (RR = 2.48; *p* = 0.027; Table 3). However, in none of the subgroups was an association between CCL2 positivity and RFS found in the univariate or multivariate analyses.

### 2.3. Association of CCL2 Protein Expression in ICs and Survival

**Figure 3:**
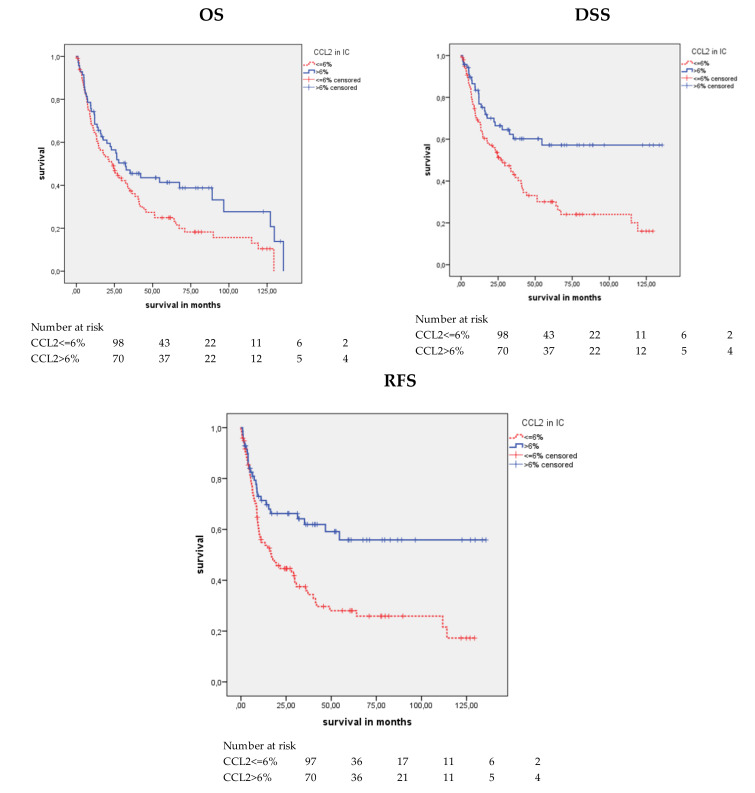
Kaplan-Meier analysis: Association between CCL2 expression in ICs and prognosis. Positive CCL2 expression in ICs was associated with a longer mean OS (P = 0.032), mean DSS (P = 0.001) and mean RFS (P = 0.001) than negative CCL2 expression.

For statistical survival analysis, an optimized cut-off for the CCL2-positive percentage was calculated by ROC analysis. The cut-off for OS, DSS, and RFS analysis was 6% (≤6% vs. >6%). For a better description, we named the groups with ≤6% CCL2 staining CCL2 negative and the group with >6% CCL2 staining CCL2 positive. A significant association with mean OS (*p* = 0.032), mean DSS (*p* = 0.001), and mean RFS (*p* = 0.001) was observed by Kaplan–Meier analysis (Table 4 and Figure 3). When comparing the patients with CCL2-positive ICs with those with CCL2-negative ICs, the mean OS was 58.0 months vs. 40.0 months, the mean DSS was 84.4 months vs. 47.2 months, and the mean RFS was 82.6 vs. 43.8 months. Univariate Cox’s regression analysis revealed that CCL2 negativity was associated with a 1.50-fold increased risk of death (*p* = 0.033; Table 5), a 2.1-fold increased risk of disease-specific death (*p* = 0.002; Table 5), and a 2.1-fold increased risk of relapse (*p* = 0.001; Table 5). Multivariate Cox’s regression analysis (adjusted for tumor stage, lymph node stage, and molecular subtype) showed that CCL2 staining was an independent predictor of DSS (RR = 1.77; *p* = 0.031; Table 5) but not for OS or RFS. Altogether, in contrast to TCs, CCL2 positivity appears to be a good prognostic factor in ICs. Since this result for the ICs is somewhat unexpected, we present the subgroup analysis for the association of CCL2 positivity with prognosis (OS, DSS, and RFS) in more detail than that for the TCs.

### 2.4. Association of CCL2 Protein Expression in ICs and Survival Stratified by Clinicopathological Parameters

#### 2.4.1. CCL2 Protein Expression and Survival in the pT2 and pT3+4 Groups

**Figure 4:**
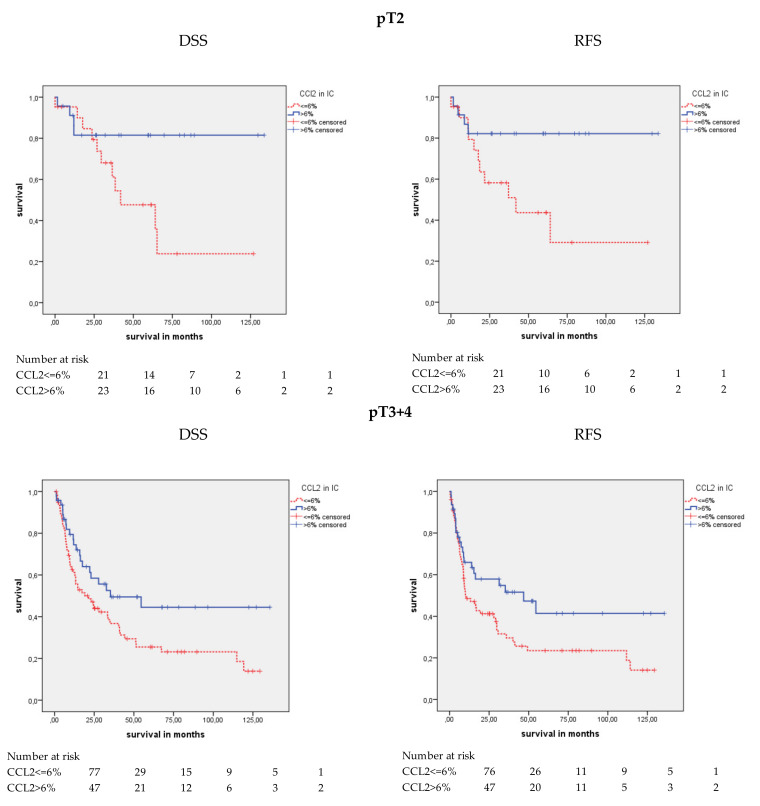
Kaplan-Meier analysis: Association of CCL2 expression stratified by tumor stage (pT2 vs. pT3+4) in ICs and prognosis. Analysis of patients in the pT2 group revealed that positive CCL2 expression was associated with longer mean DSS (*p* = 0.033) and mean RFS (*p* = 0.022) than negative CCL2 expression. Comparably for patients in the pT3+4 group, CCL2 expression was associated with longer mean DSS (*p* = 0.030) and mean RFS (*p* = 0.034).

Next, we were interested in determining whether there were differences in prognosis between the two tumor stage groups (pT2 vs. pT3+4) that were associated with CCL2 staining in IC in the Kaplan–Meier analysis (Table 4; Figure 4). There was no difference in OS in either tumor stage group for CCL2 staining. However, patients in the pT2 (*p* = 0.033) and the pT3+4 (*p* = 0.030) groups showed significant differences in DSS. Comparably, a significant difference was also observed for patients in the pT2 (*p* = 0.022) and the pT3+4 (*p* = 0.034; Table 4) groups in terms of RFS. In the pT2 group, univariate Cox’s regression analysis showed a 3.28-fold (*p* = 0.043; Table 5) increased risk for disease-specific death and a 3.51-fold increased risk for relapse (*p* = 0.032) in the CCL2-negative patients compared to the CCL2-positive patients. In the pT3+4 group, a 1.73-fold higher risk of disease-specific death and a 1.70-fold increased risk for relapse (*p* = 0.032 and *p* = 0.036) were detected in the CCL2-negative patients compared to the CCL2-positive patients. Multivariate Cox’s regression analysis revealed an association between CCL2 staining and RFS in the pT2 group (RR = 5.53; *p* = 0.020; Table 5).

#### 2.4.2. CCL2 Protein Expression and Survival in the pN0 and pN1+2 Groups

**Figure 5:**
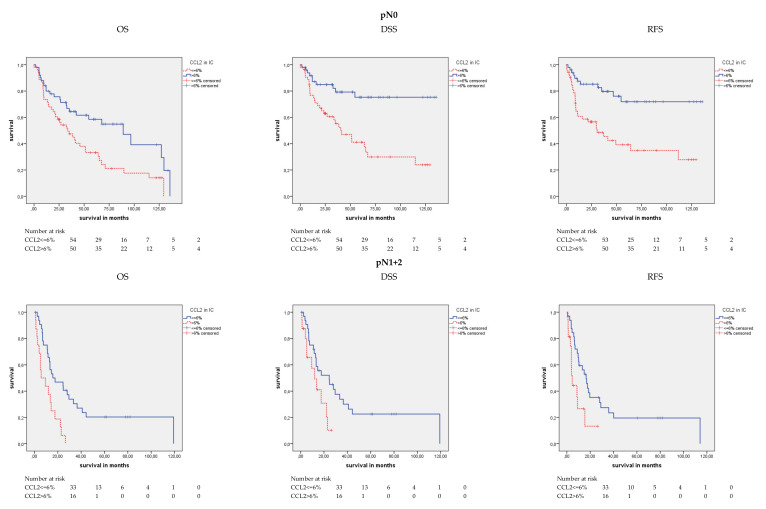
Kaplan-Meier analysis: Association of CCL2 expression stratified by lymph node stage; (N0 vs. N1+2) in ICs and prognosis. In the N0 group, CCL2-positive patients showed a longer mean OS (*p* = 0.005), mean DSS and mean RFS (both *p* < 0.001) than CCL2-negative patients. However, in the N1+2 group, CCL2-positive patients had a shorter mean OS (*p* = 0.001), mean DSS (*p* = 0.031) and RFS (*p* = 0.013).

Furthermore, differences in prognosis between the two nodal stage groups (N0 vs. N1+2) for CCL2 staining were studied (Figure 5). Interestingly, we found that CCL2 positivity was differently associated with OS, DSS, and RFS in the N0 and N1+2 groups. In the Kaplan–Meier analysis considering the N0 group, the CCL2-positive patients showed a longer OS (77.3 months) than the CCL2-negative patients (47.8 months) (*p* = 0.005; Table 4). However, in the N1+2 group, the CCL2-positive patients had a shorter OS (10.4 months) than the CCL2-negative patients (38.1 months) (*p* = 0.001; Table 4). Comparably, in the N0 group, the CCL2-positive patients had a DSS of 107.6 months and an RFS of 104.4 months, while the CCL2-negative patients had a shorter DSS of 57.9 months and a shorter RFS of 56.3 months (both *p* < 0.001; Table 4). However, in the N1+2 group, the CCL2-positive patients had a DSS of 13.2 months and an RFS of 8.8 months, whereas the CCL2-negative patients had a longer DSS of 41.1 months and a longer RFS of 34.1 months (*p* = 0.031 and *p* = 0.013; Table 4). In the univariate Cox’s regression analysis in the N0 group, patients with negative CCL2 staining showed a 2.07-fold increased risk of death, a 3.50-fold increased risk of disease-specific death, and a 3.37-fold increased risk of relapse (*p* = 0.006, *p* = 0.001, and *p* = 0.001; Table 5) compared to CCL2-positive patients. In the N1+2 group, patients with positive CCL2 staining showed a 3.02-fold increased risk of death, a 2.28-fold increased risk of disease-specific death, and a 2.56-fold increased risk of relapse (*p* = 0.001, *p* = 0.036, and *p* = 0.016; Table 5).

In the multivariate Cox’s regression analysis (adjusted to tumor stage and molecular subtype) in the N0 group, patients with negative CCL2 staining had a 1.99-fold increased risk of death, a 3.17-fold increased risk of disease-specific death, and a 3.10-fold increased risk for relapse (*p* = 0.014, *p* = 0.002, and *p* = 0.002; Table 5) compared to the patients with CCL2-positive staining. In the multivariate Cox’s regression analysis (adjusted to tumor stage and molecular subtype) in the N1+2 group, patients with positive CCL2 staining carried a 3.44-fold increased risk for death and a 4.47-fold increased risk of relapse (*p* = 0.019 and *p* = 0.010; Table 5) compared to patients with CCL2-negative staining.

#### 2.4.3. CCL2 Protein Expression and Survival of Patients Treated without/with Adjuvant Chemotherapy

**Figure 6:**
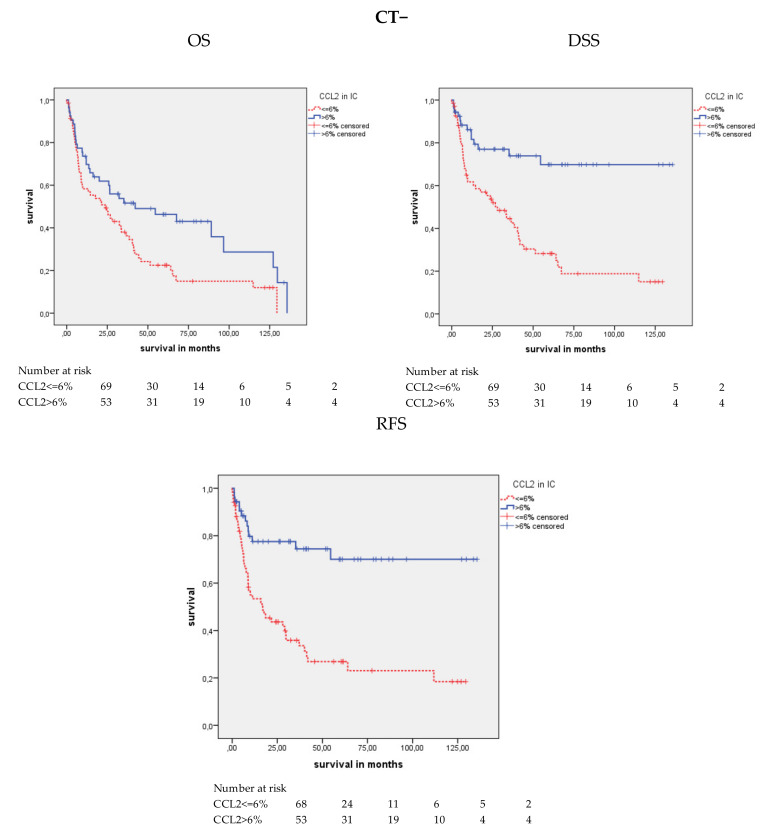
Kaplan-Meier analysis: Association of CCL2 expression with ICs and prognosis in the patient group not treated with chemotherapy. In the chemotherapy untreated (CT–) group, CCL2 positivity was positively associated with OS (*p* = 0.012), DSS (*p* < 0.001) and RFS (*p* < 0.001). However, there was no association between CCL2 staining and OS, DSS or RFS in the chemotherapy-treated group (CT+).

Patients were separated into those not treated (CT−) or treated with adjuvant chemotherapy (CT+). The Kaplan–Meier analysis revealed that CCL2 positivity was positively associated with OS (*p* = 0.012), DSS (*p* < 0.001), and RFS (*p* < 0.001) in the CT− group but not with any survival in the CT+ group (Table 4; Figure 6). Patients with CCL2 positivity showed a mean OS of 61.9 months, a DSS of 99.8 months, and an RFS of 99.8 months, whereas CCL2-negative patients had a mean OS of 37.7 months, a mean DSS of 43.0 months, and an RFS of 41.5 months. In the univariate Cox’s regression analysis, CT− patients with CCL2 negativity possessed a 1.76-fold increased risk of death (*p* = 0.013), a 3.45-fold increased risk of disease-specific death (*p* < 0.001), and a 3.59-fold increased risk of relapse (*p* < 0.001; Table 5). In the multivariate Cox’s regression analysis (adjusted for tumor stage, lymph node stage, and molecular subtype), CCL2 staining was not associated with OS, but CT− patients with CCL2 negative staining possessed a 2.44-fold increased risk of disease-specific death (*p* = 0.010) and a 2.46-fold increased risk of relapse (*p* = 0.009) compared to CCL2-positive patients (Table 5). Altogether, CCL2 staining appeared to be an independent prognostic factor for DSS and RFS in the CT− patient group.

#### 2.4.4. CCL2 Protein Expression and Survival of Patients Considering both Lymph Node Stage and Chemotherapy

Since nodal stage and chemotherapy are not independent from each other, we considered them together in a multivariate Cox’s regression interaction model and analyzed the association of CCL2 positivity with prognosis. We changed the model assumptions to analyze the interactions in the eight different groups with predefined interactions (Table 6). The eight groups arose from two groups of CCL2 staining (≤6% vs. >6%), two groups of lymph node stages (N0 vs. N1+2), and two groups of chemotherapy (without vs. with), which resulted in 2 × 2 × 2 = 8 groups. However, the groups are very small, and therefore, interpretation is limited. Considering the ≤6% vs >6% CCL2 staining groups, it was again visible that they showed a different association with OS, DSS, and RFS depending on the lymph node status, i.e., higher staining was positively associated with prognosis in the N0 group but negatively associated in the N1+2 group. Considering the lymph node status, although as expected, the N1+2 group had a worse prognosis than the N0 group, CCL2 staining could still be distinguished in each lymph node group of patients with a better or a worse prognosis. The application of chemotherapy reduced the risk of death (in OS) in the N1+2 group but not in the N0 group independent of CCL2 staining. The application of chemotherapy had no effect on the risk of tumor-associated death (DSS) independent of the lymph node stage. However, the application of chemotherapy had a controversial effect on the occurrence of relapse (RFS) in the N1+2 group dependent on CCL2 staining, i.e., in patients that received chemotherapy, RFS was increased in the >6% CCL2 staining group, but RFS was reduced in the ≤6% CCL2 staining group. Again, in the N0 group, chemotherapy had no effect on RFS independent of CCL2 staining. Altogether, for the N0 patients, chemotherapy did not show an improved OS, DSS, or RFS; however, for the N1+2 patients, OS was improved. Altogether, CCL2 staining can help to distinguish between a better and a worse prognosis in patients in all groups.

### 2.5. Association of CCL2 Protein Expression in ICs with Survival for Different Molecular Markers

#### 2.5.1. CCL2 Protein Expression and Survival of BCa Patients in the Molecular Subtypes

**Figure 7:**
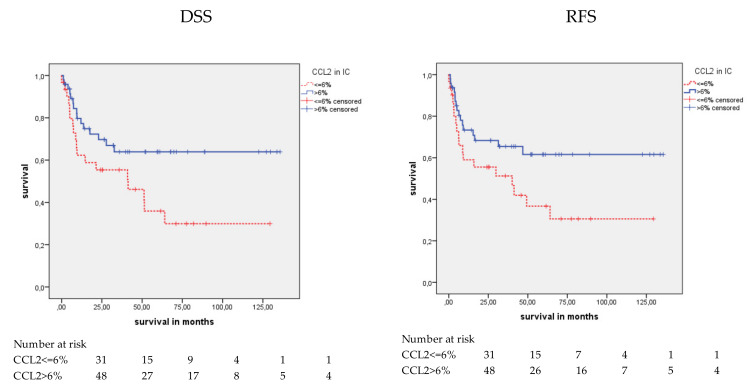
Kaplan-Meier analysis: Association of CCL2 expression in the basal subtype in ICs and prognosis. In the basal subtype, CCL2 positive staining was associated with a better mean DSS (*p* = 0.032) and mean RFS (*p* = 0.044) than CCL2 negative staining. However, no association between CCL2 staining and prognosis was found in the other molecular subtypes.

Patients were separated into four molecular subtypes, i.e., basal (*N* = 79); luminal (*N* = 69); luminal EMT-p53, such as (*N* = 16); and double negative for basal and luminal markers (*N* = 4). After molecular subtyping, CCL2 staining was not associated with OS in any subtype in the Kaplan–Meier analysis. Only in the basal subtype was CCL2 positivity associated with better DSS (*p* = 0.032) and RFS (*p* = 0.044) in the Kaplan-Meier analysis (Table 4; Figure 7). In the univariate Cox’s regression analysis, CCL2-negative patients had a 2.08-fold increased risk of disease-specific death and a 1.97-fold increased risk of relapse (*p* = 0.036 and *p* = 0.048; Table 5). In the multivariate Cox’s regression analysis (adjusted for tumor stage and lymph node stage), CCL2-negative patients showed a 2.27-fold increased risk of disease-specific death (*p* = 0.029; Table 5). In summary, CCL2 staining appeared to be an independent prognostic factor for DSS in the basal subtype.

#### 2.5.2. Correlation of sTILs and CCL2-Expressing Immune Cells

A large part of the ICs are stromal tumor-infiltrating lymphocytes (sTILs). When applying a cut-off percentage of 6% for both sTILs and CCL2, a strong overlap (79.7%) between both ≤6% cell populations was found (Supplementary Table S3). However, in the tumors with >6% sTILs, approximately half had a CCL2 percentage ≤6% (46.8%), and the other half had an IC CCL2 percentage >6% (53.2%).

This presents the question are the >6% sTILs with >6% CCL2 different from those with ≤6% CCL2 staining?

To answer this question, we performed survival analysis in terms of the percentage of sTILs in all patients and stratified the patients by their nodal stage (N0 vs. N1+2).

#### 2.5.3. Association of Percentage of sTILs with Survival

As expected, a higher presence of sTILs (>6% vs. ≤6%) was significantly associated with increased OS, DSS, and RFS in all patients (all *p*<0.001) (Supplementary Figure S1). However, when stratifying patients by their nodal status (N0 vs. N1+2), a higher presence of sTILs (>6%) was only significantly associated with longer OS, DSS, and RFS in the N0 patient group (all *p* < 0.001; Supplementary Figure S1), but no association was found in the N1+2 group.

Another possibility is that macrophages are immune cells that could show different CCL2 expression depending on the nodal stage. To determine if this was the case, we performed survival analysis of CD68 staining as a pan macrophage marker and CD163 as an M2 macrophage marker in all patients and stratified the patients by their nodal stage (N0 vs. N1+2).

#### 2.5.4. Correlation of CD68- and CCL2-Expressing Immune Cells

Macrophages are a large component of the IC population. After applying a cut-off for CD68 staining, the median (>median [>10.4] vs. ≤median [≤10.4]), and a cut-off for CCL2 (6% of CCL2 stained ICs), there was a strong overlap (79.3%) between the low CCL2 (≤6%) and the low CD68 (≤median) staining groups (Supplementary Table S4) In the group with high CD68 (<median) staining, as expected, a small percentage of cells showed low CCL2 (38.4%), and a high percentage showed high CCL2 (61.6%) staining.

#### 2.5.5. Association of CD68 Staining with Survival

Higher CD68 staining (>median vs. ≤median) was associated with better DSS and RFS (*p* = 0.041; *p* = 0.028; log rank test; Supplementary Figure S2). However, when stratifying patients by their nodal stage (N0 vs. N1+2), in the N0 patient group, high CD68 staining was only significantly associated with longer DSS and RFS (*p* = 0.023 and *p* = 0.016; log rank test; Supplementary Figure S2) than that with low CD68 staining, but no association was found in the N1+2 group.

#### 2.5.6. Correlation of CD163- and CCL2-Expressing Immune Cells

After applying a cut-off for CD163 staining, the median (>median [10.34] vs. ≤median [≤10.34]), and a cut off for CCL2 (6% of CCL2 stained ICs), there was again an overlap (72.6%) between the low CCL2 (≤6%) and the low CD68 (≤median) staining groups (Supplementary Table S5). In the group with high CD163 (>median) staining, a smaller percentage of cells showed low CCL2 (44.0%) and a higher percentage showed high CCL2 (56.0%). However, we did not see a difference in the numbers of ICs expressing CD136 (>median) between the N0 and N1+2 patient groups.

#### 2.5.7. Association of CD163 Staining with Survival

CD163 staining (>median vs. ≤median) was not associated with prognosis (OS, DSS, and RFS) in all patients. When stratifying patients by their nodal stage (N0 vs. N1+2), in the N1+2 group, high CD163 staining was significantly associated with a shorter OS (*p*=0.016, log rank test, Supplementary Figure S3), as a trend with DSS (*p* = 0.086) but not with RFS and no association was found in the N0 group.

The results for the sTILs, macrophages, and the above results suggest that CCL2 can act as a marker in the N1+2 group in a subpopulation of immune cells (>6% CCL2 percentage) that is associated with a shorter DSS and RFS and in a subpopulation of immune cells (≤6% CCL2 percentage) that is associated with a longer DSS and RFS. However, these ICs are not restricted to only one type of IC, such as sTILs or macrophages.

Another possibility could be that PD-L1 expression on TCs or ICs could further characterize CCL2-expressing ICs.

#### 2.5.8. Correlation of PD-L1-Expressing TCs or ICs and CCL2-Expressing Immune Cells

We applied the median as the cut-off for PD-L1 staining of TCs (0 vs. >0) and ICs (≤1 vs. >1). A strong correlation between low PD-L1-expressing TCs or ICs and low CCL2-expressing ICs and between PD-L1-expressing TCs or ICs and high CCL2-expressing ICs was detected (*p* < 0.001; Supplementary Tables S6 and S7).

#### 2.5.9. Association of PD-L1 Expression with Survival

PD-L1 expression in TCs did not reveal any association with OS, DSS, or RFS. PD-L1 expression on ICs (>1) showed an association with better OS, DSS, and RFS (all *p* < 0.001; Supplementary Figure S4). However, again stratifying patients by their nodal stage (N0 vs. N1+2), compared to lower PD-L1 staining in the N0 patient group, higher PD-L1 staining was significantly associated with longer OS, DSS, and RFS (*p*<0.001; Supplementary Figure S4), but no association was found in the N1+2 group.

## 3. Discussion

We analyzed, for the first time, the protein expression of CCL2 in TCs and ICs in BCa patients (N = 168) and assessed its association with clinicopathological and survival data. The expression of CCL2 in TCs was considered positive or negative, and expression in the ICs was considered as the percentage of CCL2-expressing cells of all ICs; this scoring system resulted in a negative group with ≤6% CCL2-expressing ICs and a positive group with >6% CCL2-expressing ICs. CCL2 staining in the TCs was not correlated with staining in the ICs. Remarkably, CCL2 staining in the TCs was positively correlated with the age at cystectomy and negatively correlated with OS and time to recurrence. In contrast, CCL2 staining of ICs was negatively correlated with tumor stage, lymph node stage, and molecular subtype and positively correlated with OS, DSS, RFS, and CK5 staining. In addition, as expected, CCL2 staining of the ICs was significantly positively correlated with the presence of different immune cells/cell markers, such as CD3 (T cells), CD8 (T cells), CD68 (macrophages), PD-L1 in TCs and ICs, and the percentage of stromal tumor-infiltrating lymphocytes (sTILs).

The role of CCL2 in cancers is still controversial [25,26]. Mostly, the detection of CCL2 in tumors is considered a marker for tumor progression/metastasis, e.g., in cancers of the breast, prostate, colon, and thyroid glands [28,29,30,31,33]. Our finding of a correlation of CCL2 staining with lymph node status supports previous results, as CCL2 expression is correlated with lymph node metastasis in papillary thyroid carcinoma [29]. On the other hand, CCL2 production confirmed in neoplastic ducts of the pancreas could be a relevant negative regulator of pancreatic cancer progression [35]. Furthermore, serum CCL2 levels are positively correlated with tumor macrophage infiltration in pancreatic cancers [35].

We showed for the first time that CCL2 expression in TCs is an independent negative predictor for OS in muscle invasive BCa patients. Comparably, patients with diffuse large B cell lymphoma (DLBCL) and a higher CCL2 expression had a shorter OS and progression-free survival [40]. Breast cancer patients with high CCL2 expression in their tumor cells possessed a shorter RFS [27], and for clear cell renal cell cancer patients, elevated CCL2 expression was correlated with clinical stage, OS, and macrophage infiltration [32]. However, NSCLC patients with higher CCL2 expression showed a significantly better OS [37]. Furthermore, in line with our findings, another CC chemokine ligand (CCL18) has been shown to play a critical role in the progression of bladder cancer [41]. After stratifying our patients’ cohort further, we found that CCL2 positivity in TCs was an independent negative prognostic factor for OS for patients with pT2 stage, with chemotherapy treatment, and with the luminal subtype

Furthermore, we described for the first time that the expression of CCL2 in ICs is generally a positive independent prognostic factor for DSS in muscle invasive BCa patients. After molecular stratification, in the basal subtype, positive CCL2 staining in the ICs remained a positive independent prognostic factor for DSS. A role of CCL2 mRNA in the basal subtype has been described in a breast cancer mouse model recently; however, tumor-associated macrophages induce CCL2 expression in tumor cells [42].

Remarkably, after stratifying the BCa patients according to their lymph node stage (N0 vs. N1+2), CCL2 staining in ICs gave contradictory results for prognosis. For N0 patients, CCL2 staining was a positive independent prognostic factor for OS, DSS, and RFS. However, for N1+2 patients, CCL2 staining was a negative independent prognostic factor for OS and RFS. Such a contradictory effect for CCL2 in ICs according to lymph node status has not yet been described in tumors. When testing whether sTILs, macrophages, and PD-L1 in ICs all show a similar behavior, we observed an association between sTILs, macrophages, and PD-L1 on ICs with a good prognosis in N0 patients, but we did not see an association between sTILs, macrophages or PD-L1 in ICs and prognosis in N1+2 patients. Our result that sTILs and PD-L1 expression are associated with a good prognosis in BCa is in accordance with the literature [19,20,43]. The finding that macrophages are associated with a good prognosis in BCa patients is controversial in the literature [19]. However, the results may depend on cut-offs and on the application of different antibodies and they are not easily comparable (Eckstein et al. 2019). In addition, the ICs expressing CCL2 may comprise different ICs [25], e.g., macrophages have been classified as classical M1 (antitumor macrophages) and alternative M2 (protumor macrophages) polarized subtypes [26]. Since CD68 is a pan-macrophage marker, a more M2-specific macrophage marker, i.e., CD163 can be applied [44]. In a mouse tumor model from a breast metastasis cell line (MDA-MB-231), stimulation of CCL2 expression by M2 macrophages was recently shown [42]. Recently, Xu et al. could show that increased levels of CCL2 polarize macrophages in multiple myelomas toward the M2-like phenotype that generally suppresses antitumor immunity [45]. They suggest as a mechanism that CCL2 upregulates the expression of the immunosuppressive molecular MCP-1-induced protein (MCPIP1) in macrophages [45]. However, we did not see differences in the number of M2-like macrophages (CD163 positive) between N0 and N1+2 patients. Possibly, CCL2 could rather affect macrophages qualitatively than quantitatively.

Our findings suggest that CCL2 could mark a population of ICs that may be antitumorigenic in N0 but protumorigenic in N1+2 muscle invasive BCa patients. This fits the general thinking that a tumor, such as bladder cancer, itself is a key immunological player that can shape immune responses to favor itself [44]. Another aspect is that high-grade BCa tumors could recruit more mast cells, which are also ICs, to produce CCL2 via estrogen receptor β signaling [46]. However, mast cells were not characterized in this study.

In addition, when comparing patients receiving chemotherapy or not, high CCL2 expression in ICs was associated with a better prognosis in patients without chemotherapy, but here no association of CCL2 with prognosis was observed as high CCL2 expression in TC was associated with a shorter OS and DSS in patients receiving a chemotherapy. Although, we cannot compare patients treated with chemotherapy with those not treated with chemotherapy since the tumor biology and prognosis are quite different, we saw no positive effect of the application of chemotherapy in the N0 patients, at least in our patient cohort. Recently, a study of muscle invasive BCa in Sweden showed that patients treated with radio/chemotherapy or radiotherapy had a better OS and DSS than untreated patients [47]. However, treated patients were not further stratified by their lymph node status. Thus, we think that before suggesting chemotherapy to muscle invasive BCa patients, the lymph node stage should be considered. In addition, an effect of chemotherapy on the presence of ICs might also be considered. However, Waidhauser et al. showed that chemotherapy markedly reduces B cells but not T cells and natural killer cells in cancer patients [48]. The future aim is to develop tumor therapies that kill cancer cells without harming the cells that constrain tumor growth, particularly T cells and other immune cells [49]. A possible way could be the application of glutamine antagonists that affect tumor cells but not immune cells [49,50].

Our study has some limitations. This was a retrospective study, and for a comprehensive statistical analysis of three parameters in eight groups, the number of study patients was too low. In addition, only 27.4% (47/168) of our patients were treated with chemotherapy, which is again a rather small cohort. Therefore, our results must be evaluated in a larger prospective study. However, altogether, the number of study patients (*n* = 168) allowed reasonable multivariate analysis of the association of one parameter, such as CCL2 staining, with prognosis in muscle invasive BCa patients.

Altogether, CCL2 positivity in TCs is a negative prognostic factor for OS. For ICs, the prognosis depends on the lymph node status, i.e., CCL2 can mark ICs associated with good prognosis in N0 patients, whereas it marks ICs associated with poor prognosis in N1+2 patients. Altogether, CCL2 staining on TCs and on ICs is suggested as a prognostic biomarker for muscle invasive BCa patients.

## 4. Material and Methods

### 4.1. Patients and Tumor Material

Tissue microarrays (TMAs) with formalin-fixed and paraffin-embedded tumor samples from 168 muscle invasive BCa patients were investigated in this study. The TMA was prepared as follows: Hematoxylin and eosin stained slides were scanned (Panoramic P250, 3DHistech, Budapest, Hungary) and annotated using a TMA annotation tool (Caseviewer v2). Four cores (diameter 1 mm; two cores from the invasion margin, two cores from the tumor center) were taken utilizing an automated tissue microarrayer (TMA Grandmaster, 3DHistech, Budapest, Hungary) as described previously [43,51]. The research carried out on human subjects is in compliance with the Helsinki Declaration. All patients gave written informed consent. The study is based on the approval of the Ethics Commission of the University Hospital Erlangen (No. 3755 and No. 329_16B). Tumor histology was reviewed by two uropathologists (AH, ME). An overview of the clinicopathological parameters of the patients included in this study is given in Table 1.

### 4.2. Immunohistochemistry

For the study of CCL2 protein expression, a manual IHC protocol was applied as previously described [52]. Briefly, after heat pretreatment at 120 °C for 5 min with TE–buffer pH 9 and peroxidase blocking (Dako, Hamburg, Germany), primary antibody against CCL2 (monoclonal mouse IgG1, clone 2D8, Cat.-No. AM06749PU-N, dilution 1:15,000; Acris Antibodies, Herford, Germany) was applied for 30 min. The slides were counterstained for 1 min with hematoxylin (Merck, Darmstadt, Germany). Between all of the steps, the slides were washed with buffer from Dako, and all of the incubation steps were performed at room temperature. For the study of the other proteins, staining was performed on a fully automated Ventana Benchmark Ultra autostainer (Ventana, Tucson, AZ, USA). IHC staining was performed with the following antibodies: CK5 (monoclonal mouse IgG, clone XM26; dilution 1:50; Diagnostic BioSystems, Pleasanton, CA, USA), GATA3 (monoclonal mouse IgG, clone L50-823; dilution 1:100; Sigma-Aldrich, Taufkirchen, Germany), CD3 (monoclonal mouse IgG, clone F7.2.38, ThermoFisher-Scientific, Darmstadt, Germany, dilution 1:50), CD8 (mouse monoclonal IgG, clone C8/144B ThermoFisher-Scientific, dilution 1:50), CD68 (mouse monoclonal IgG, clone PG-M1, ThermoFisher-Scientific, dilution 1:60), CD163 (mouse moncolonal IgG, clone 10D6, Novocastra/Leica, Wetzlar, Germany, dilution 1:500), and PD-L1 (SP263 assay, Ventana, Vreden, Germany). PD-L1+ immune cells (ICs) and tumor cells (TCs) were scored by two pathologists (AH, ME) according to the distributor’s PD-L1 scoring algorithm as previously described [43]. Briefly, sections were deparaffinized, and antigens were retrieved by heating the sections in a pH 8.4 Tris/borate/EDTA solution (Ventana). Endogenous peroxidase was blocked with 1% H_2_O_2_. Visualization of bound antibody was performed using the ultraVIEW TM DAB system (Ventana). All sections were counterstained with hematoxylin II/Mayer’s hematoxylin (Ventana).

Stained specimens were viewed at objective magnifications of ×100 and ×200. Negative control slides without the addition of primary antibody were included for each staining experiment. From each sample, two cores from the center and two cores from the invasive front were analyzed. Afterwards, the staining average of both cores was determined since we did not see significant differences between both locations.

The expression of CCL2 was detected in TCs (average of stained TCs in the invasion front and in the tumor center) and characterized as positive or negative and in ICs (average of stained ICs in the invasion front and the tumor center) as the percentage of CCL2-positive ICs out of all ICs. There were no relevant differences in staining intensities so only those positive or negative for the TC and only the percentage of CCL2-positive IC were counted. PD-L1 staining was determined as the percentage of stained TCs or ICs. Tumor-infiltrating lymphocytes were assessed as described previously [24,53]. For the survival analysis, patients were grouped as CCL2 positive vs. negative in TCs and ≤6% CCL2 cells vs. >6% CCL2-positive ICs. Furthermore, for the survival analysis, patients were grouped according to the median percentage of PD-L1 staining in TCs (0% vs. >0%) or in ICs (≤1% vs. >1%). For the survival analysis, patients were also grouped by the median (**≤**median vs. >median) of the log2 transformed expression for CD3, CD8, and CD68. Slides were scanned with a P250 slide scanner (3DHistech, Budapest, Hungary) and analyzed using CaseViewer2.0 (3DHistech). Photos were taken with a Leica DM 4000B microscope with a 20× HC PL Fluotar objective (Leica, Wetzlar, Germany) and with a Jenoptik Gryphax Arktur camera (Jenoptik AG, Jena, Germany).

### 4.3. Immune Cell Quantification via Definiens Developer Software

CD3+, CD8+, and CD68+ ICs were quantified (counts per mm^2^) and log2-transformed for further analysis with Definiens Developer Software as described previously [24].

### 4.4. Molecular Subtyping via NanoString Technology

RNA was isolated and purified as described previously [24]. We selected 21 genes, which are known as stable markers for luminal and basal differentiation, according to the MDDACC subtyping approach [7,9,24]. Gene counts were normalized using two reference genes (SDHA, HPRT1) and log2-transformed for further analysis with nSolver 4.0 software.

### 4.5. Statistical Analyses

The associations between the IHC and clinicopathological data were calculated using the Spearman’s correlation test, the Chi-squared test, or the Mann–Whitney test. The associations of the expression of CCL2 with OS, DSS, and RFS were determined in univariate analyses (Kaplan–Meier analysis and Cox’s regression hazard models) and in multivariate Cox’s regression analyses. Multivariate Cox’s regression analyses were adjusted for parameters that were significantly associated with prognosis in univariate Cox’s regression analysis, i.e., tumor stage, lymph node stage, and molecular subtype (Supplementary Table S8). A *p*-value of less than 0.05 was considered statistically significant. Statistical analyses were performed with the SPSS 21.0 software package (SPSS Inc., Chicago, IL, USA).

## 5. Conclusions

Positive CCL2 staining in TCs was an independent negative prognostic factor for OS in muscle invasive BCa patients. In contrast, positive CCL2 staining in ICs appeared to be a positive independent prognostic factor for DSS in muscle invasive BCa. Most intriguingly, after separating the patients according to their lymph node status (N0 vs. N1+2), CCL2 staining in the ICs was contrarily associated with prognosis. In the N0 group, CCL2 positivity in ICs was a positive independent prognostic factor for OS, DSS, and RFS, whereas in the N1+2 group, CCL2 positivity was a negative independent factor for OS, DSS, and RFS in multivariate Cox’s regression analyses. In summary, CCL2 staining on TCs and on ICs is suggested as a prognostic biomarker for muscle invasive BCa patients.

## Figures and Tables

**Table 1:** Clinicopathological data and survival parameters of the BCa patients.

Clinicopathological and Survival Parameters	Patients (Percentage)
Total	168
**Morphology**	
NOS	91 (54.1)
Squamous	37 (22.0)
Sarcomatoid	9 (5.4)
MPUC	9 (5.4)
PUC	6 (3.6)
Pure neuroendocrine	4 (2.4)
Other rare subtypes	12 (7.1)
**Gender**	
female	42 (25)
male	126 (75)
**Age (years)**	
range	41.0-90.0
mean	69.7
median	71.0
**Tumor Stage**	
pT2	44 (26.2)
pT3	85 (50.6)
pT4	39 (23.2)
**Tumor Grade 1973**	
G2	6 (3.6)
G3	162 (96.4)
**Tumor Grade 2016**	
high grade	168
**Nodal Stage**	
pN0	104 (61.9)
pN1+2	49 (29.2)
pNX	15 (8.9)
**Adjuvant Chemotherapy (Ct)**	
yes	46 (27.4)
no	122 (72.6)
**Survival/observation Time (months)**	
range	0-135.7
mean	34.5
median	23.9
**Overall Survival (OS)**	
alive	46 (27.4)
dead	122 (72.6)
**Disease-Specific Survival (DSS)**	
alive	78 (46.4)
dead	90 (53.6)
**Relapse-Free Survival Time (months)**	
range	0-135.7
mean	31.0
median	16.1
**Relapse-Free Survival (RFS)**	
without relapse	92 (54.8)
with relapse	76 (45.2)
**Stromal TILs**	
0	11 (6.6)
1%–9%	58 (34.5)
10%–50%	84 (50.0)
51%–80%	13 (7.7)
>80%	2 (1.2)
**Molecular Subtypes**	
Basal	79 (47.0)
DN	4 (2.4)
Luminal	69 (41.1)
Luminal EMT-p53-like	16 (9.5)

Abbreviations: DN = double negative for basal and luminal markers; TILs = tumor-infiltrating lymphocytes; OS = overall survival; DSS = disease-specific survival; RFS = relapse-free survival, NOS = not other specified; MPUC = micropapillary urothelial carcinoma, PUC = plasmacytoid urothelial carcinoma.

**Table 2:** Kaplan–Meier analysis: Association of CCL2 staining in TCs with mean OS, mean DSS, or mean RFS.

Parameter			Kaplan–Meier Analysis					
**CCL2**	**N**	**OS**		**DSS**		**N**	**RFS**	
positive vs. negative								
		**Months**	***p***	**Months**	***p***		**Months**	***p***
**All Patients**	168	33.6 vs. 55.4	**0.004**	49.9 vs. 67.9	**0.036**	167	48.3 vs. 65.2	**0.047**
**Tumor Stage 2**	44	29.8 vs. 78.6	**0.003**	n.s.	n.s.	44	n.s.	n.s.
**Tumor Stage 3+4**	124	n.s.	n.s.	n.s.	n.s.	123	n.s.	n.s.
**Nodal Stage N0**	104	45.1 vs. 71.2	**0.016**	n.s.	n.s.	103	n.s.	n.s.
**Nodal Stage N1/N2**	49	n.s.	n.s.	n.s.	n.s.	49	n.s.	n.s.
**Nodal Stage NX**	15	n.s.	n.s.	n.s.	n.s.	15	n.s.	n.s.
**CT−**	122	35.4 vs. 56.3	**0.025**	n.s.	n.s.	121	n.s.	n.s.
**CT+**	46	25.8 vs. 51.7	**0.043**	26.4 vs. 59.3	**0.045**	46	21.4 vs. 49.6	(0.070)
**Basal**	79	n.s.	n.s.	n.s.	n.s.	79	n.s.	n.s.
**Luminal**	69	29.1 vs. 58.6	**0.010**	36.9 vs. 61.8	(0.060)	68	33.3 vs. 58.9	(0.052)
**Luminal EMT-p53-like**	16	n.s.	n.s.	22.3 vs. 54.6	(0.059)	16	15.8 vs. 54.6	(0.064)

Abbreviations: Basal—basal subtype; Luminal—Luminal subtype; Luminal EMT-p53—Luminal EMT-p53 subtype; CT—adjuvant chemotherapy; n.s.—not significant.

**Table 3:** Univariate and multivariate Cox’s regression analysis: Association of CCL2 staining in TCs with mean OS, mean DSS, or mean RFS.

Parameter			Univariate Cox’s Regression Analysis					
**CCL2**	**N**	**OS**		**DSS**		**N**	**RFS**	
positive vs. negative								
		**RR**	***p***	**RR**	***p***		**RR**	***p***
**All Patients**	168	1.69	**0.005**	1.57	**0.037**	167	1.53	**0.047**
**Tumor Stage 2**	44	3.28	**0.003**	n.s.	n.s.		n.s.	n.s.
**Tumor Stage 3+4**	124	n.s.	n.s.	n.s.	n.s.	123	n.s.	n.s.
**Nodal Stage N0**	104	1.83	0.018	n.s.	n.s.	103	n.s.	n.s.
**Nodal Stage N1/N2**	49	n.s.	n.s.	n.s.	n.s.	49	n.s.	n.s.
**Nodal Stage NX**	15	n.s.	n.s.	n.s.	n.s.	15	n.s.	n.s.
**CT−**	122	1.62	**0.026**	n.s.	n.s.	121	n.s.	n.s.
**CT+**	46	2.05	**0.048**	2.10	**0.050**	46	1.94	(0.075)
**Basal**	79	n.s.	n.s.	n.s.	n.s.	79	n.s.	n.s.
**Luminal**	69	2.05	**0.012**	1.77	(0.064)	68	1.80	(0.056)
**Luminal EMT-p53-like**	16	n.s.	n.s.	n.s.	n.s.	16	n.s.	n.s.
			**Multivariate Cox’s Regression Analysis**					
**CCL2**	**N**	**OS**		**DSS**		**N**	**RFS**	
positive vs. negative								
		**RR**	***p***	**RR**	***p***		**RR**	***p***
**All Patients**	168	1.70	**0.007**	1.52	(0.068)		1.48	(0.081)
**Tumor Stage 2**	44	4.02	**0.002**	n.s.	n.s.	44	n.s.	n.s.
**Tumor Stage 3+4**	124	n.s.	n.s.	n.s.	n.s.	123	n.s.	n.s.
**Nodal Stage N0**	104	1.65	(0.059)	n.s.	n.s.	103	n.s.	n.s.
**Nodal Stage N1/N2**	49	1.99	(0.054)	1.90	(0.098)	49	1.92	(0.086)
**Nodal Stage NX**	15	n.s.	n.s.	n.s.	n.s.	15	n.s.	n.s.
**CT−**	122	1.49	(0.087)	n.s.	n.s.	122	n.s.	n.s.
**CT+**	46	2.56	0.018	2.48	**0.027**	46	2.09	(0.060)
**Basal**	79	1.76	(0.059)	2.00	(0.062)	79	1.85	(0.097)
**Luminal**	69	1.97	**0.021**	n.s.	n.s.	68	1.76	(0.074)
**Luminal EMT-p53-like**	16	n.s.	n.s.	n.s.	n.s.	16	n.s.	n.s.

Abbreviations: Basal—basal subtype; Luminal—Luminal subtype; Luminal EMT-p53—Luminal EMT-p53 subtype; CT—adjuvant chemotherapy; n.s.—not significant; significant *p*-values are marked in bold face.

**Table 4:** Kaplan–Meier analysis: Association of CCL2 staining in ICs with mean OS, mean DSS, or mean RFS.

Parameter			Kaplan–Meier Analysis					
**CCL2 percentage**	**N**	**OS**		**DSS**		**N**	**RFS**	
<=6% vs. >6%								
		**Months**	***p***	**Months**	***p***		**Months**	***p***
**All Patients**	168	40.0 vs. 58.0	**0.032**	47.2 vs. 84.4	**0.001**	167	43.8 vs. 82.6	**0.001**
**Tumor Stage 2**	44	n.s.	n.s.	59.7 vs. 110.6	**0.033**	44	57.3 vs. 111.1	**0.022**
**Tumor Stage 3+4**	124	n.s.	n.s.	43.3 vs. 70.6	**0.030**	123	39.2 vs. 66.3	**0.034**
**Nodal Stage N0**	104	47.8 vs. 77.3	**0.005**	57.9 vs. 107.6	**<0.001**	103	56.3 vs. 104.4	**<0.001**
**Nodal Stage N1/N2**	49	38.1 vs. 10.4	**0.001**	41.1 vs. 13.2	**0.031**	49	34.1 vs. 8.8	**0.013**
**Nodal Stage NX**	15	n.s.	n.s.	n.s.	n.s.	15	n.s.	n.s.
**CT−**	122	37.7 vs. 61.9	**0.012**	43.0 vs. 99.8	**<0.001**	121	41.5 vs. 99.8	**<0.001**
**CT+**	46	n.s.	n.s.	n.s.	n.s.	46	n.s.	n.s.
**Basal**	79	n.s.	n.s.	54.9 vs. 91.3	**0.032**	79	53.5 vs. 88.3	**0.044**
**Luminal**	69	n.s.	n.s.	n.s.	n.s.	68	n.s.	n.s.
**Luminal EMT-p53-like**	16	n.s.	n.s.	n.s.	n.s.	16	n.s.	n.s.

Abbreviations: Basal—basal subtype; Luminal—Luminal subtype; Luminal EMT-p53—Luminal EMT-p53 subtype; CT—adjuvant chemotherapy; n.s.—not significant; significant values are in bold.

**Table 5:** Univariate and multivariate Cox’s regression analysis: Association of CCL2 staining in ICs with mean OS, mean DSS, or mean RFS.

Parameter			Univariate Cox’s Regression Analysis					
**CCL2 percentage**	**N**	**OS**		**DSS**		**N**	**RFS**	
<=6% vs. >6%								
		**RR**	***p***	**RR**	***p***		**RR**	***p***
**All Patients**	168	1.50	**0.033**	2.10	**0.002**	167	2.10	**0.001**
**Tumor Stage 2**	44	n.s.	n.s.	3.28	**0.043**	44	3.51	**0.032**
**Tumor Stage 3+4**	124	n.s.	n.s.	1.73	**0.032**	123	1.70	**0.036**
**Nodal Stage N0**	104	2.07	**0.006**	3.50	**0.001**	103	3.37	**0.001**
**Nodal Stage N1/N2**	49	3.02	**0.001**	2.28	**0.036**	49	2.56	**0.016**
**Nodal Stage NX**	15	n.s.	n.s.	n.s	n.s.	15	n.s.	n.s.
**CT−**	122	1.76	**0.013**	3.45	**<0.001**	121	3.59	**<0.001**
**CT+**	46	n.s.	n.s.	n.s.	n.s.	46	n.s.	n.s.
**Basal**	79	n.s.	n.s.	2.08	**0.036**	79	1.97	**0.048**
**Luminal**	69	n.s.	n.s.	n.s.	n.s.	68	n.s.	n.s.
**Luminal EMT-p53-like**	16	n.s.	n.s.	n.s.	n.s.	16	n.s.	n.s.
			**Multivariate Cox’s Regression Analysis**					
**CCL2 percentage**	**N**	**OS**		**DSS**		**N**	**RFS**	
<=6% vs. >6%								
		**RR**	***p***	**RR**	***p***		**RR**	***p***
**All patients**	168	n.s.	n.s.	1.77	**0.031**	167	n.s.	n.s.
**Tumor Stage 2**	44	n.s.	n.s.	n.s.	n.s.	44	5.53	**0.020**
**Tumor Stage 3+4**	124	n.s.	n.s.	n.s.	n.s.	123	n.s.	n.s.
**Nodal Stage N0**	104	1.99	**0.014**	3.17	**0.002**	103	3.10	**0.002**
**Nodal Stage N1/N2**	49	3.44	**0.019**	3.06	(0.062)	49	4.47	**0.010**
**Nodal Stage NX**	15	n.s.	n.s.	n.s.	n.s.	15	n.s.	n.s.
**CT−**	122	n.s.	n.s.	2.44	**0.010**	121	2.46	**0.009**
**CT+**	46	n.s.	n.s.	n.s.	n.s.	46	n.s.	n.s.
**Basal**	79	n.s.	n.s.	2.27	**0.029**	79	n.s.	n.s.
**Luminal**	69	n.s.	n.s.	n.s.	n.s.	68	n.s.	n.s.
**Luminal EMT-p53-like**	16	n.s.	n.s.	n.s.	n.s.	16	n.s.	n.s.

Abbreviations: Basal—basal subtype; Luminal—Luminal subtype; Luminal EMT-p53—Luminal EMT-p53 subtype; CT—adjuvant chemotherapy; n.s.—not significant; significant values are in bold.

**Table 6:** Multivariate Cox’s regression analysis: Interactions of the parameters CCL2 staining, lymph node status, and application of chemotherapy.

**OS**	**Lymph Node**	**Chemotherapy**	**N**	**RR**	***p***
**CCL2 Percentage**					
>6%	N1+2	no	10	13.43	<0.001
>6%	N1+2	yes	6	6.67	0.008
≤6%	N1+2	no	18	4.69	0.014
≤6%	N1+2	yes	15	2.11	n.s.
>6%	N0	no	42	1.12	n.s.
>6%	N0	yes	8	N.A.	
≤6%	N0	no	41	2.10	n.s.
≤6%	N0	yes	13	3.05	n.s. (0.088)
**DSS**	**Lymph Node**	**Chemotherapy**	**N**	**RR**	***p***
**CCL2 Percentage**					
>6%	N1+2	no	10	6.39	0.012
>6%	N1+2	yes	6	6.24	0.011
≤6%	N1+2	no	18	1.62	n.s.
≤6%	N1+2	yes	15	1.66	n.s.
>6%	N0	no	42	0.42	n.s.
>6%	N0	yes	8	N.A.	
≤6%	N0	no	41	1.65	n.s.
≤6%	N0	yes	13	2.42	n.s.
**RFS**	**Lymph Node**	**Chemotherapy**	**N**	**RR**	***p***
**CCL2 Percentage**					
>6%	N1+2	no	10	3.75	n.s. (0.052)
>6%	N1+2	yes	6	8.03	0.001
≤6%	N1+2	no	18	3.33	0.033
≤6%	N1+2	yes	15	1.41	n.s.
>6%	N0	no	42	0.31	n.s. (0.059)
>6%	N0	yes	8	N.A.	
≤6%	N0	no	41	1.32	n.s.
≤6%	N0	yes	13	1.89	n.s.

N.A. = not analyzed because all patients survived; n.s. = not significant.

## Supplementary Materials

The following are available online at https://www.mdpi.com/2072-6694/12/5/1253/s1, Figure S1: Kaplan–Meier analysis: Association of TiLs and prognosis in all patients and in N0 patients, Figure S2: Kaplan–Meier analysis: Association of macrophages (CD68) and prognosis in all patients and in N0 patients, Figure S3 Kaplan–Meier analysis: Association of M2-like macrophages (CD163) and OS/DSS in N1+2 patients, Figure S4: Kaplan–Meier analysis: Association of PD-L1 in IC with prognosis in all patients and in N0 patients, Table S1: CCL2 expression in TCs and in ICs of muscle invasive BCa patients, Table S2: Spearman’s bivariate correlation between CCL2 staining in TCs or ICs and clinicopathological/molecular parameters in BCa patients, Table S3: Cross Table: Correlation of CCL2 staining and TIL percentage, Table S4: Cross Table: Correlation of CCL2 staining and CD68 (pan macrophage marker), Table S5: Cross Table: Correlation of CCL2 staining and CD163 (M2-like macrophage marker), Table S6: Cross Table: Correlation of CCL2 staining and PD-L1 in TCs, Table S7: Cross Table: Correlation of CCL2 staining and PD-L1 in ICs, Table S8: Univariate Cox’s regression analysis: Association of clinicopathological and molecular parameters with prognosis.

## Abbreviations

CCL2	CC-chemokine-ligand 2
BCa	bladder cancer
OS	overall survival
DSS	disease-specific survival
RFS	relapse free survival
TCs	tumor cells
ICs	immune cells
IHC	immunohistochemistry
CD3	CD3 antigen
CD8	CD8 antigen
PD-L1	Programmed death ligand 1 (CD274 molecule)
IL1B	Interleukin 1B
EMT	Epithelial Mesenchymal Transition
SDHA	Succinate dehydrogenase complex subunit A
HPRT1	Hypoxanthine guanine phosphoribosyltransferase 1
