# Supplementary material for: CCL2 Expression in Tumor Cells and Tumor-Infiltrating Immune Cells Shows Divergent Prognostic Potential for Bladder Cancer Patients Depending on Lymph Node Stage
Source: Cancers (Basel). 2020 May 15;12(5):1253. doi: 10.3390/cancers12051253 (PMC7281525; doi:10.3390/cancers12051253)
Supplement: Supplementary file 1 [file cancers-12-01253-s001.pdf]

Supplementary Materials: CCL2 Expression in Tumor Cells and Tumor-Infiltrating Immune Cells Shows Divergent Prognostic Potential for Bladder Cancer Patients Depending on Lymph Node Stage

Markus Eckstein, Elena Epple, Rudolf Jung, Katrin Weigelt, Verena Lieb, Danijel Sikic, Robert Stöhr, Carol Geppert, Veronika Weyerer, Simone Bertz, Astrid Kehlen, Arndt Hartmann, Bernd Wullich, Helge Taubert and Sven Wach

All patients

OS

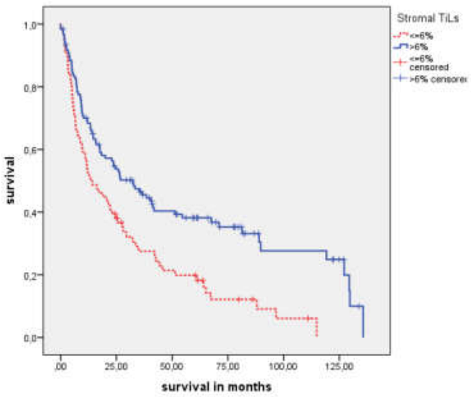

Number at risk						
CCL2<=6%	76	28	14	6	2	0
CCL2>6%	122	61	38	22	10	6

DSS

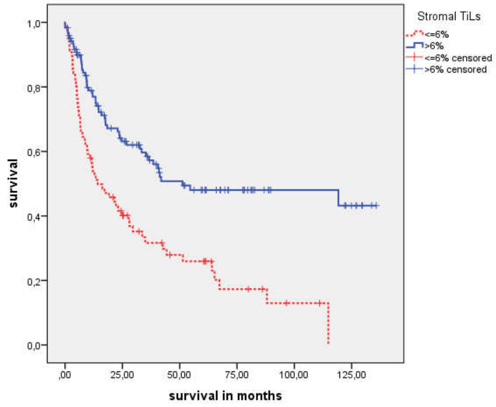

Number at risk						
CCL2<=6%	76	28	14	6	2	0
CCL2>6%	122	61	38	22	10	6

RFS

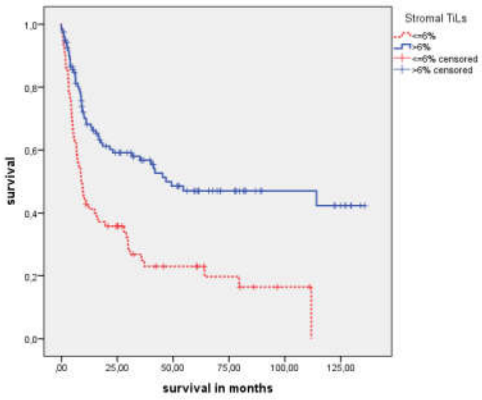

Number at risk						
CCL2<=6%	75	24	10	6	2	0
CCL2>6%	122	56	35	20	10	6

N0 patients

OS

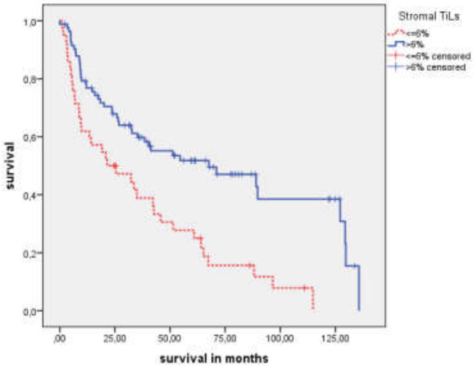

Number at risk						
CCL2<=6%	42	20	11	5	2	0
CCL2>6%	83	52	34	18	9	6

DSS

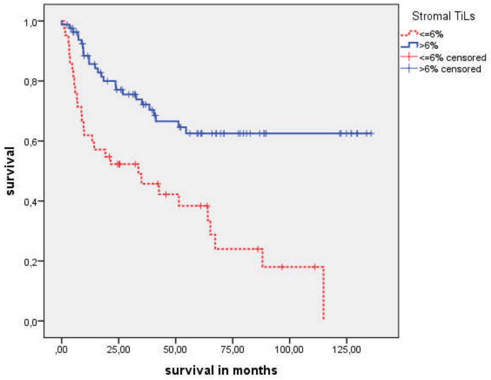

Number at risk						
CCL2<=6%	42	20	11	5	2	0
CCL2>6%	83	52	34	18	9	6

RFS

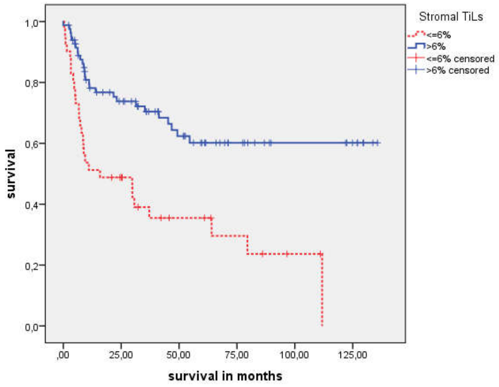

Number at risk						
PD-L1<=1%	41	18	8	5	2	0
PD-L1>1%	83	49	31	16	9	6

Figure S1. Kaplan–Meier analysis: Association of TiLs and prognosis in all patients and in N0 patients.

All patients

DSS

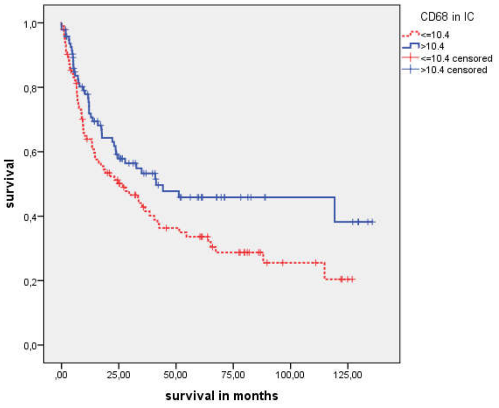

Number at risk						
CD68<=10.4	103	45	27	17	6	1
CD68>10.4	95	44	25	11	6	5

RFS

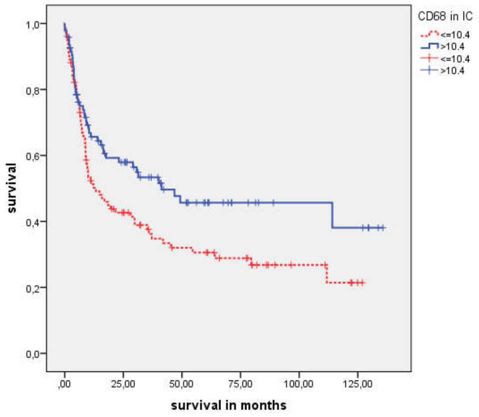

Number at risk						
CD68<=10.4	102	37	22	16	6	1
CD68>10.4	95	43	23	10	6	5

N0 patients

DSS

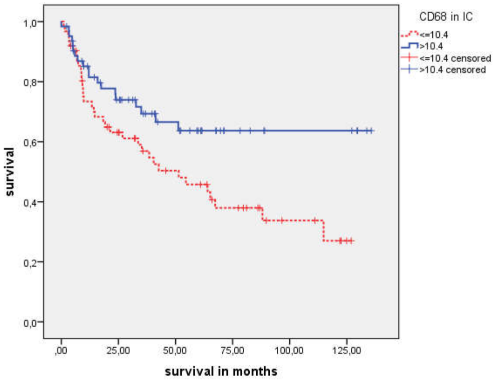

Number at risk						
CD68<=10.4	62	34	22	14	6	1
CD68>10.4	63	38	23	9	5	5

RFS

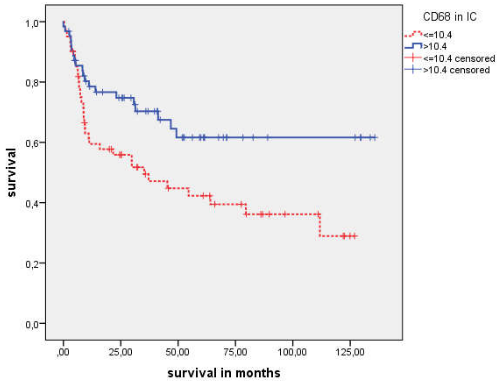

Number at risk						
CD68<=10.4	61	29	18	13	6	1
CD68>10.4	63	38	21	8	5	5

Figure S2. Kaplan–Meier analysis: Association of macrophages (CD68) and prognosis in all patients and in N0 patients.

N1+2 patients

OS

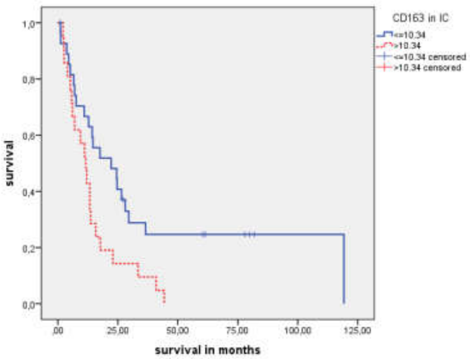

Number at risk						
CD163<=10.34	28	11	6	4	1	0
CD163>10.34	21	3	0	0	0	0

DSS

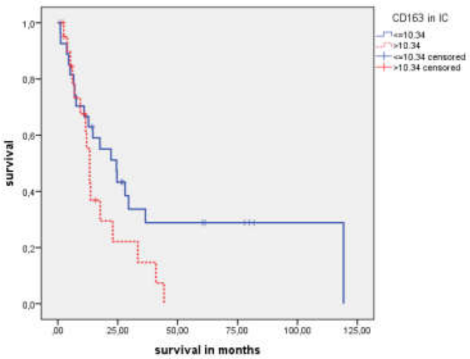

Number at risk						
CD163<=10.34	28	11	6	4	1	0
CD163>10.34	21	3	0	0	0	0

Figure S3. Kaplan–Meier analysis: Association of M2-like macrophages (CD163) and OS/DSS in N1+2 patients.

All patients

OS

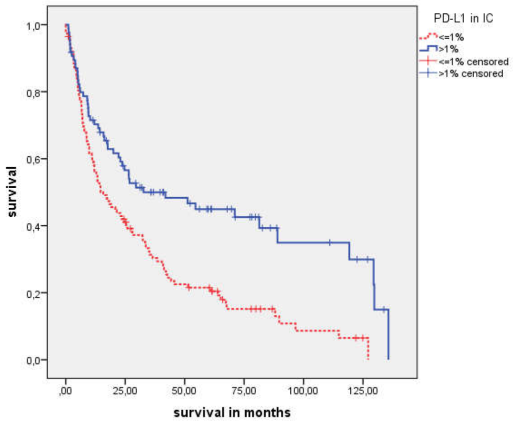

Number at risk

PD-L1<=1%	113	45	23	11	4	1
PD-L1>1%	85	44	29	17	8	5

DSS

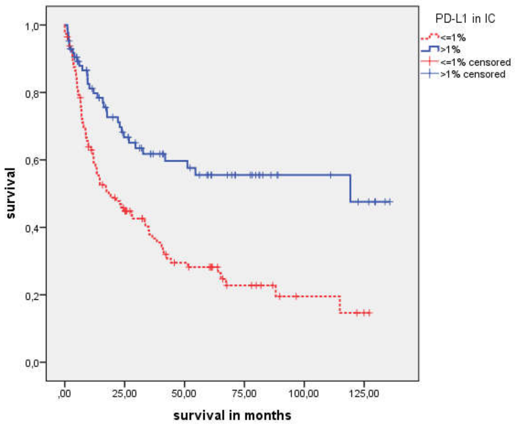

Number at risk

PD-L1<=1%	113	45	23	11	4	1
PD-L1>1%	85	44	29	17	8	5

RFS

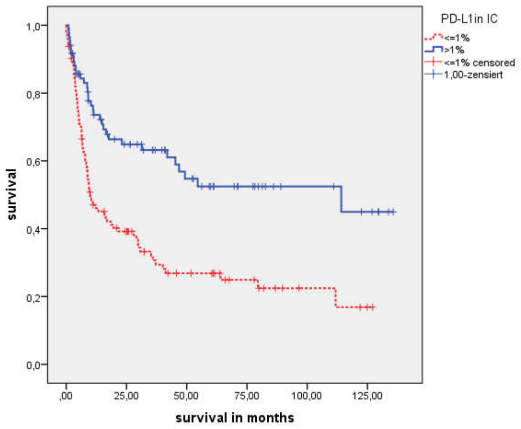

Number at risk

PD-L1<=1%	112	38	19	11	4	1
PD-L1>1%	85	42	26	15	8	5

N0 patients

OS

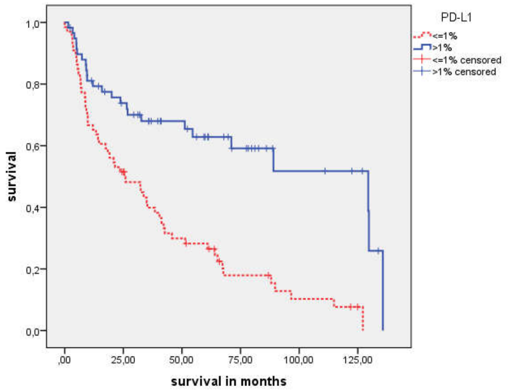

Number at risk

PD-L1<=1%	66	33	18	8	4	1
PD-L1>1%	59	39	27	15	7	5

DSS

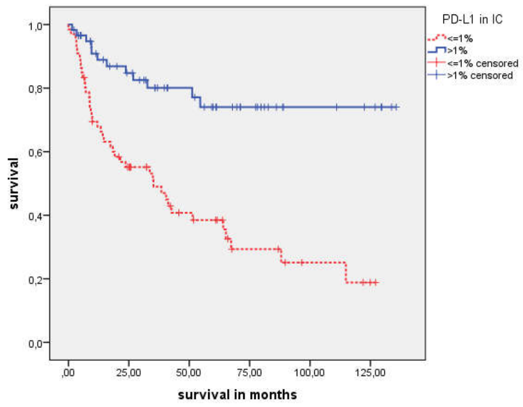

Number at risk

PD-L1<=1%	66	33	18	8	4	1
PD-L1>1%	59	39	27	15	7	5

RFS

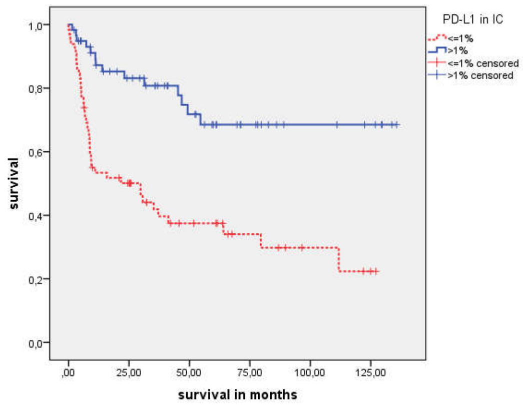

Number at risk

PD-L1<=1%	65	29	15	8	4	1
PD-L1>1%	59	38	24	13	7	5

Figure S4. Kaplan–Meier analysis: Association of PD-L1 in IC with prognosis in all patients and in N0 patients.

Table S1. CCL2 expression in TCs and in ICs of muscle invasive BCa patients.

Cells	N	CCL2 Negative (in %)	CCL2 Positive (in %)
Tumor cells (TCs)	168	111 (66.1)	57 (33.9)
Immune cells (ICs)	168	98 (58.3)	70 (41.7)

Table S2. Spearman's bivariate correlation between CCL2 staining in TCs or ICs and clinicopathological/molecular parameters in BCa patients.

Parameter		CCL2 in TCs		CCL2 in ICs
**Gender**	rs	-0.016	rs	0.004
	p	0.841	*p*	0.961
**Age at cystectomy**	rs	.240*	rs	0.005
	p	**0.002**	*p*	0.954
**pT**	rs	0.138	rs	−0.155*
	p	0.074	*p*	**0.045**
**pN**	rs	0.079	rs	−0.210*
	p	0.307	*p*	**0.006**
**OS**	rs	-.242*	rs	0.177*
	p	**0.002**	*p*	**0.022**
**DSS**	rs	-0.134	rs	0.344**
	p	0.084	*p*	**<0.001**
**time to recurrence**	rs	-.199*	rs	0.081
	p	**0.010**	*p*	0.298
**RFS**	rs	-0.117	rs	0.353**
	p	0.130	*p*	**<0.001**
**CCL2 in ICs/TCs**	rs	-0.068	rs	−0.068
	p	0.381	*p*	0.381
**CK5**	rs	-.172*	rs	0.201*
	p	**0.025**	*p*	**0.009**
**GATA3**	rs	-0.092	rs	0.125
	p	0.233	*p*	0.105
**Stromal TILs**	rs	-0.128	rs	0.559**
	p	0.096	*p*	**<0.001**
**CD3**	rs	-0.105	rs	0.604**
	p	0.174	*p*	**<0.001**
**CD8**	rs	-0.113	rs	0.591**
	p	0.144	*p*	**<0.001**
**CD68**	rs	-0.137	rs	0.500**
	p	0.074	*p*	**<0.001**
**PD-L1 (SP263) in ICs**	rs	-0.151	rs	0.541**
	p	0.051	*p*	**<0.001**
**PD-L1 (SP263) in TCs**	rs	-0.097	rs	0.351**
	p	0.212	*p*	**<0.001**
**molecular subtypes**	rs	0.017	rs	−0.336**
	p	0.819	*p*	**<0.001**

Significant values are marked in bold. rs = correlation coefficient, p = p-value (2-sided), *$p > 0.05$, **$p < 0.001$.

Table S3. Cross Table: Correlation of CCL2 staining and TIL percentage.

CCL2			Stromal TILs		Sum
			≤6%	>6%	
CCL2	≤6%	**N**	47	51	98
		% within CCL2	48.0%	52.0%	100.0%
		% within stromal TILs	79.7%	46.8%	58.3%
		% of the sum	28.0%	30.4%	58.3%
	>6%	N	12	58	70
		% within CCL2	17.1%	82.9%	100.0%
		% within stromal TILs	20.3%	53.2%	41.7%
		% of the sum	7.1%	34.5%	41.7%
All		*N*	59	109	168
		% within CCL2	35.1%	64.9%	100.0%
		% within stromal TILs	100.0%	100.0%	100.0%
		% of the sum	35.1%	64.9%	100.0%

Chi-squared test, $p < 0.001$.

Table S4. Cross Table: Correlation of CCL2 staining and CD68 (pan macrophage marker).

CCL2			CD68		Sum
			log2 median ≤10.4	log2 median >10.4	
CCL2	≤6%	**N**	65	33	98
		% within CCL2	66.3%	33.7%	100.0%
		% within CD68	79.3%	38.4%	58.3%
		% of the sum	38.7%	19.6%	58.3%
	>6%	*N*	17	53	70
		% within CCL2	24.3%	75.7%	100.0%
		% within CD68	20.7%	61.6%	41.7%
		% of the sum	10.1%	31.5%	41.7%
All		*N*	82	86	168
		% within CCL2	48.8%	51.2%	100.0%
		% within CD68	100.0%	100.0%	100.0%
		% of the sum	48.8%	51.2%	100.0%

Chi-squared test, $p < 0.001$.

Table S5. Cross Table: Correlation of CCL2 staining and CD163 (M2-like macrophage marker).

CCL2			CD163		Sum
			log2 median ≤10.34	log2 median >10.34	
CCL2	≤6%	**N**	61	37	98
		% within CCL2	62.2%	37.8%	100.0%
		% within CD163	72.6%	44.0%	58.3%
		% of the sum	38.7%	19.6%	58.3%
	>6%	*N*	23	47	70
		% within CCL2	32.9%	67.1%	100.0%
		% within CD163	27.4%	56.0%	41.7%
		% of the sum	13.7%	28.0%	41.7%
All		*N*	84	84	168
		% within CCL2	50.0%	50.0%	100.0%
		% within CD163	100.0%	100.0%	100.0%
		% of the sum	50.0%	50.0%	100.0%

Chi-squared test, $p < 0.001$.

Table S6. Cross Table: Correlation of CCL2 staining and PD-L1 in TCs.

**CCL2**			**PD-L1**		**Sum**
			0	>0	
**CCL2**	**≤6%**	*N*	72	26	98
		% within CCL2	73.5%	26.5%	100.0%
		% within PD-L1	70.6%	39.4%	58.3%
		% of the sum	42.9%	15.5%	58.3%
	**>6%**	*N*	30	40	70
		% within CCL2	42.9%	57.1%	100.0%
		% within PD-L1	29.4%	60.6%	41.7%
		% of the sum	17.9%	23.8%	41.7%
**All**		*N*	102	66	168
		% within CCL2	60.7%	39.3%	100.0%
		% within PD-L1	100.0%	100.0%	100.0%
		% of the sum	60.7%	39.3%	100.0%

Chi-squared test, $p < 0.001$.

Table S7. Cross Table: Correlation of CCL2 staining and PD-L1 in ICs.

**CCL2**			PD-L1		Sum
			≤1	>1	
**CCL2**	**≤6%**	N	74	24	98
		% within CCL2	75.5%	24.5%	100.0%
		% within PD-L1	78.7%	32.4%	58.3%
		% of the sum	44.0%	14.3%	58.3%
	>6%	*N*	20	50	70
		% within CCL2	28.6%	71.4%	100.0%
		% within PD-L1	21.3%	67.6%	41.7%
		% of the sum	11.9%	29.8%	41.7%
All		*N*	94	74	168
		% within CCL2	56.0%	44.0%	100.0%
		% within PD-L1	100.0%	100.0%	100.0%
		% of the sum	56.0%	44.0%	100.0%

Chi-squared test, $p < 0.001$.

Table S8. Univariate Cox's regression analysis: Association of clinicopathological and molecular parameters with prognosis.

		**Univariate Cox's Regression Analysis**						
	**N**	**OS**		**DSS**		***N***	**RFS**	
		RR	*p*	RR	*p*		RR	*p*
**Age**	168					167		
>71 years vs. <=71 years	87 vs. 81	1.37	0.087	1.20	0.383	86 vs. 81	1.18	0.425
**Pathol. Tumor Stage**	168					167		
pT3+4 vs pT2	124 vs. 44	1.88	**0.005**	2.40	**0.002**	123 vs. 44	2.48	**0.001**
**Pathol. Nodal Stage**	153					152		
pN0 (reference)	104	1		1		103	1	
pN1+2	49	2.48	**<0.001**	2.99	**<0.001**	49	3.08	**<0.001**
pNX	15	5.27	**<0.001**	7.02	**<0.001**	15	7.04	**<0.001**
**Chemotherapy**	168					167		

CT+ vs. CT-	46 vs. 122	1.06	0.775	1.29	0.250	121	1.48	0.074
**Molecular Subtype**	168					167		
basal (reference)	79	1		1		79	1	
double negative	4	1.82	0.249	2.23	0.184	4	2.02	0.246
luminal	69	1.21	0.343	1.56	0.055	68	1.58	**0.048**
luminal EMT-p53-like	16	1.87	**0.042**	2.14	**0.031**	16	2.18	**0.027**

Abbreviations: CT—adjuvant chemotherapy. Significant values are in bold.
